# Supplementary material for: Effect of Calcium Fortified Foods on Health Outcomes: A Systematic Review and Meta-Analysis
Source: Nutrients. 2021 Jan 22;13(2):316. doi: 10.3390/nu13020316 (PMC7911363; doi:10.3390/nu13020316)
Supplement: Supplementary file 1 [file nutrients-13-00316-s001.pdf]

Figure S1a: Calcium intake (mg/day)

Study or Subgroup	Mean Difference	SE	Weight	Mean Difference IV, Random, 95% CI
**1.26.1 RCTs -Children**				
Bass 2007	396	106	14.3%	396.00 [188.24, 603.76]
Du 2004	191.7	16.6323	30.1%	191.70 [159.10, 224.30]
Zhang 2014 (boys)	337	33.1498	27.8%	337.00 [272.03, 401.97]
Zhang 2014 (girls)	353	33.092	27.8%	353.00 [288.14, 417.86]
**Subtotal (95% CI)**			**100.0%**	**306.17 [198.97, 413.38]**
Heterogeneity: Tau² = 9665.77; Chi² = 30.65, df = 3 (P < 0.00001); I² = 90%				
Test for overall effect: Z = 5.60 (P < 0.00001)				
**1.26.2 RCTs - Adults**				
Ferrar 2011	318.107	109.31	27.5%	318.11 [103.86, 532.35]
Kukuljan 2009	675	62.27	34.4%	675.00 [552.95, 797.05]
Zhang 2016	398	28.8598	38.1%	398.00 [341.44, 454.56]
**Subtotal (95% CI)**			**100.0%**	**471.47 [266.51, 676.42]**
Heterogeneity: Tau² = 27871.25; Chi² = 17.60, df = 2 (P = 0.0002); I² = 89%				
Test for overall effect: Z = 4.51 (P < 0.00001)				
**1.26.3 RCTs - Postmenopausal Women**				
Green 2002	1,210	24.08	100.0%	1210.00 [1162.80, 1257.20]
**Subtotal (95% CI)**			**100.0%**	**1210.00 [1162.80, 1257.20]**
Heterogeneity: Not applicable				
Test for overall effect: Z = 50.25 (P < 0.00001)				
**1.26.4 Non-RCTs - Adults**				
Osler 1998	349.75	8.0156	50.4%	349.75 [334.04, 365.46]
Trautvetter 2012	934	52.5214	49.6%	934.00 [831.06, 1036.94]
**Subtotal (95% CI)**			**100.0%**	**639.57 [67.03, 1212.11]**
Heterogeneity: Tau² = 169262.66; Chi² = 120.93, df = 1 (P < 0.00001); I² = 99%				
Test for overall effect: Z = 2.19 (P = 0.03)				
**1.26.5 Non-RCTs - Postmenopausal Women**				
Gonzalez Sanchez 2012	103.1	3.57	100.0%	103.10 [96.10, 110.10]
**Subtotal (95% CI)**			**100.0%**	**103.10 [96.10, 110.10]**
Heterogeneity: Not applicable				
Test for overall effect: Z = 28.88 (P < 0.00001)				
Test for subgroup differences: Chi² = 2092.14, df = 4 (P < 0.00001), I² = 99.8%				

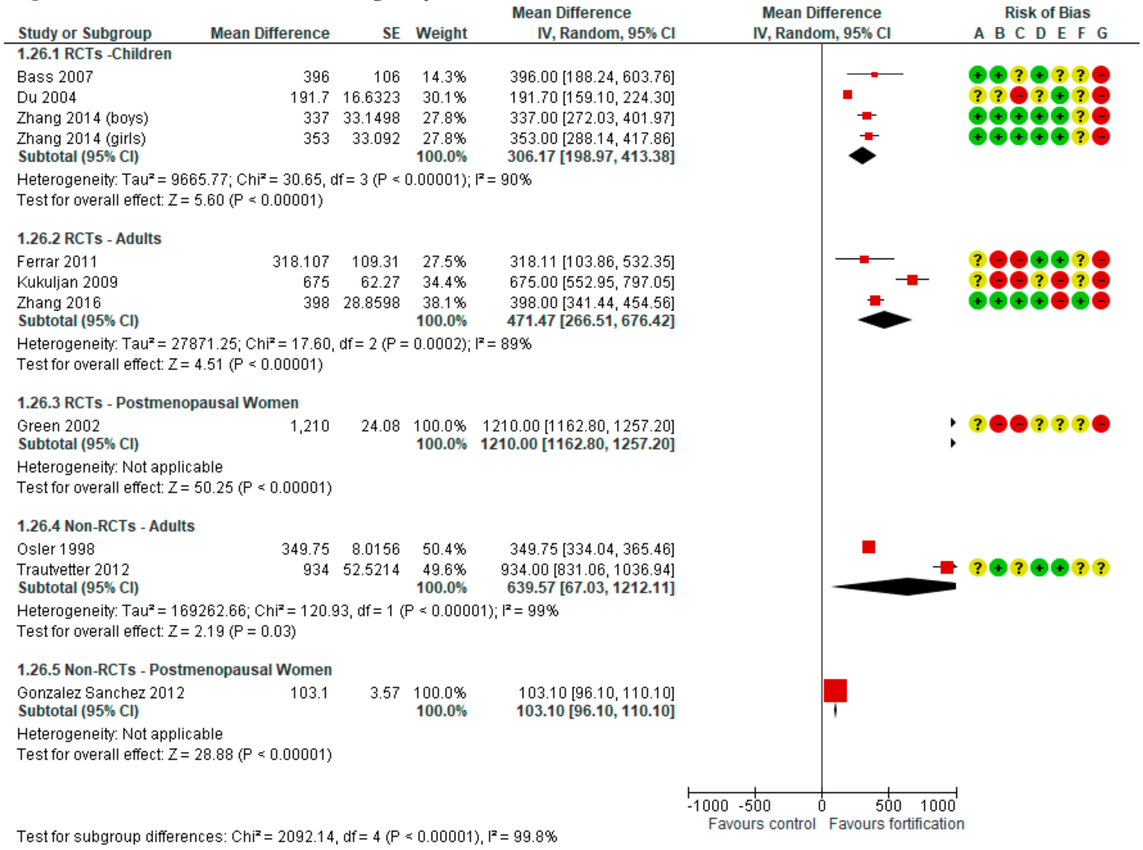

Figure S1b: Calcium intake by calcium fortification level (mg/day) reported in RCTs

Study or Subgroup	Fortification Mean	Fortification SD	Fortification Total	Control Mean	Control SD	Control Total	Weight	Mean Difference IV, Random, 95% CI
**1.42.1 Low fortification 244 mg/day**								
Ferrar 2011	948.4	242.7	19	873.5	442.3	20	100.0%	74.90 [-147.55, 297.35]
**Subtotal (95% CI)**			**19**			**20**	**100.0%**	**74.90 [-147.55, 297.35]**
Heterogeneity: Not applicable								
Test for overall effect: Z = 0.66 (P = 0.51)								
**1.42.2 Medium fortification 459-600 mg/day**								
Ferrar 2011	1,171.2	327	17	873.5	442.3	20	2.5%	297.70 [49.23, 546.17]
Zhang 2014 (boys)	949	145	38	729	135	36	33.4%	220.00 [156.20, 283.80]
Zhang 2014 (girls)	901	184	36	655	126	37	26.6%	246.00 [173.47, 318.53]
Zhang 2016	1,067	171	50	769	131	50	37.5%	298.00 [238.29, 357.71]
**Subtotal (95% CI)**			**141**			**143**	**100.0%**	**258.09 [218.64, 297.54]**
Heterogeneity: Tau² = 153.05; Chi² = 3.29, df = 3 (P = 0.35); I² = 9%								
Test for overall effect: Z = 12.82 (P < 0.00001)								
**1.42.3 High fortification 676-900 mg/day**								
Ferrar 2011	1,440	284.8	20	873.5	442.3	20	3.4%	566.50 [335.95, 797.05]
Zhang 2014 (boys)	1,194	192	35	729	135	36	30.6%	465.00 [387.60, 542.40]
Zhang 2014 (girls)	1,110	210	38	655	126	37	30.0%	455.00 [376.86, 533.14]
Zhang 2016	1,267	222	50	769	131	50	35.9%	498.00 [426.55, 569.45]
**Subtotal (95% CI)**			**143**			**143**	**100.0%**	**477.35 [434.53, 520.17]**
Heterogeneity: Tau² = 0.00; Chi² = 1.31, df = 3 (P = 0.73); I² = 0%								
Test for overall effect: Z = 21.85 (P < 0.00001)								
Test for subgroup differences: Chi² = 60.63, df = 2 (P < 0.00001), I² = 96.7%								

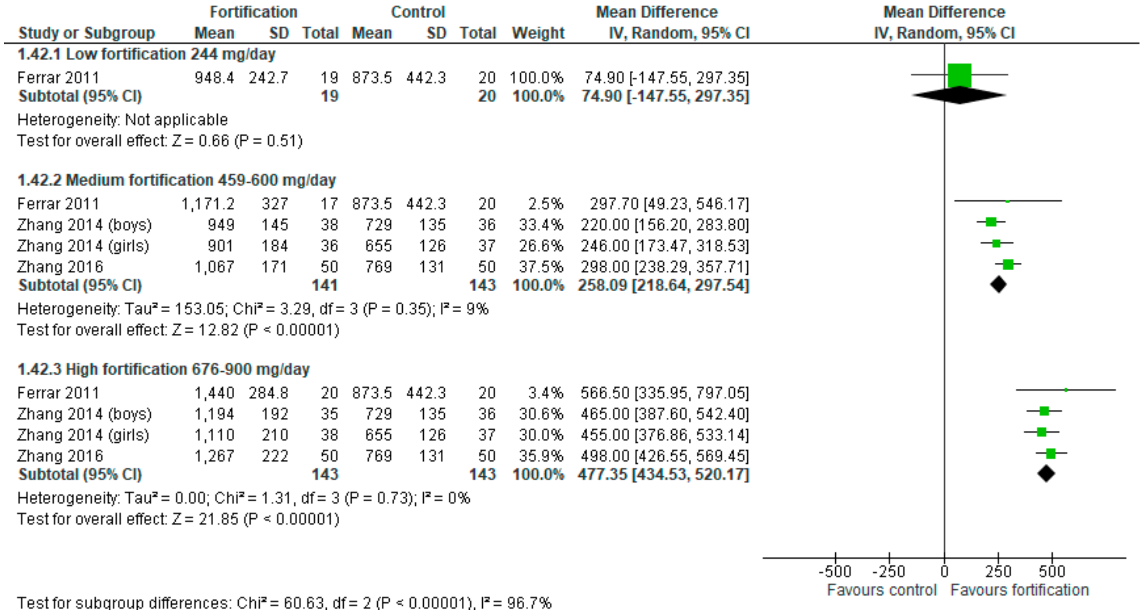

Figure S2: Weight (kg)

	Fortification			Control				Mean Difference
**Study or Subgroup**	**Mean**	**SD**	**Total**	**Mean**	**SD**	**Total**	**Weight**	**IV, Random, 95% CI**
**1.3.1 Children**								
Bass 2007	31.008	4.5917	21	29.76	4.0874	26	21.4%	1.25 [-1.27, 3.76]
Bonjour 1997	30.4	6.261	80	30.6	5.8146	69	36.0%	-0.20 [-2.14, 1.74]
Cheng 2005	48.507	7.31	49	49.59	7.21	48	16.2%	-1.08 [-3.97, 1.81]
Merrilees 2002	38.4	8.6023	74	39	10.7331	80	14.5%	-0.60 [-3.66, 2.46]
Zhang 2014 (boys)	58.38	15.893	73	53.2	16.9	36	3.1%	5.18 [-1.44, 11.80]
Zhang 2014 (girls)	49.508	10.114	74	48.1	9.9	37	8.8%	1.41 [-2.53, 5.34]
**Subtotal (95% CI)**			**371**			**296**	**100.0%**	**0.22 [-0.95, 1.38]**
Heterogeneity: Tau² = 0.00; Chi² = 4.39, df = 5 (P = 0.50); I² = 0%								
Test for overall effect: Z = 0.36 (P = 0.72)								
**1.3.2 Adults**								
Zhang 2016	52.75	9.779	100	50.9	7.3	50	100.0%	1.85 [-0.94, 4.64]
**Subtotal (95% CI)**			**100**			**50**	**100.0%**	**1.85 [-0.94, 4.64]**
Heterogeneity: Not applicable								
Test for overall effect: Z = 1.30 (P = 0.19)								
**1.3.3 Postmenopausal Women (Vit D cointervention in both groups)**								
Palacios 2005	66.89	9.04	39	66.92	9.44	40	100.0%	-0.03 [-4.11, 4.05]
**Subtotal (95% CI)**			**39**			**40**	**100.0%**	**-0.03 [-4.11, 4.05]**
Heterogeneity: Not applicable								
Test for overall effect: Z = 0.01 (P = 0.99)								

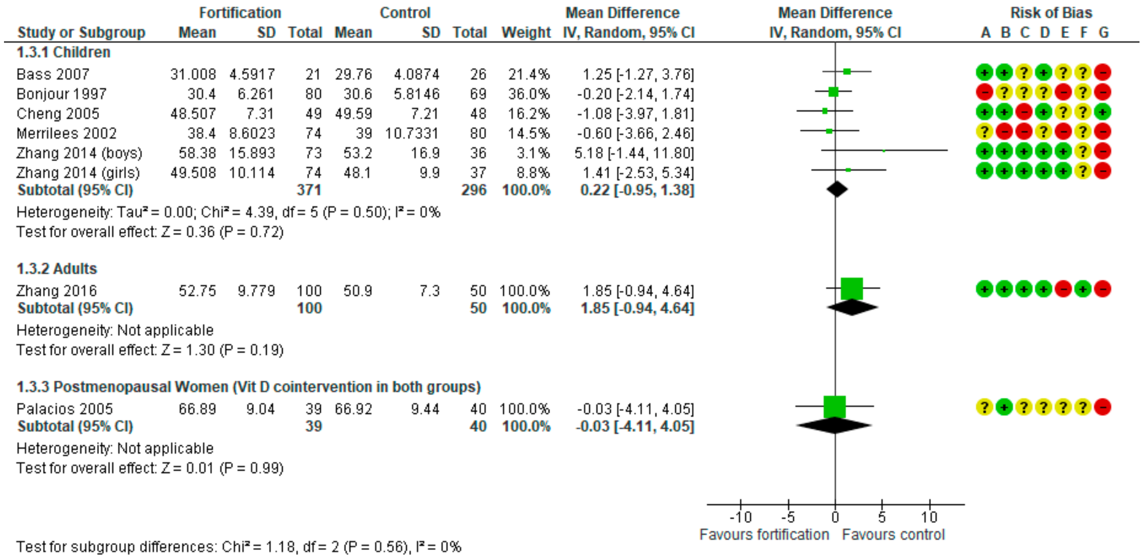

Test for subgroup differences: Chi² = 1.18, df = 2 (P = 0.56), I² = 0%

Figure S3: Height (cm)

	Fortification			Control				Mean Difference
**Study or Subgroup**	**Mean**	**SD**	**Total**	**Mean**	**SD**	**Total**	**Weight**	**IV, Random, 95% CI**
**1.4.1 Children**								
Bass 2007	140.45	5.5266	21	138.36	5.6242	26	6.6%	2.09 [-1.11, 5.29]
Bonjour 1997	134.1	8.0498	80	132.2	6.6453	69	12.2%	1.90 [-0.46, 4.26]
Cheng 2005	157.1	8.3	49	158.7	7.52	48	6.8%	-1.60 [-4.75, 1.55]
Du 2004	153.8	6.5	238	152.9	6.2	259	54.3%	0.90 [-0.22, 2.02]
Merrilees 2002	144.3	8.6023	74	145.3	8.9443	80	8.8%	-1.00 [-3.77, 1.77]
Zhang 2014 (boys)	167.64	13	73	166.7	11.2	36	3.1%	0.94 [-3.78, 5.66]
Zhang 2014 (girls)	159.11	7.37	74	157.4	7.3	37	8.1%	1.71 [-1.18, 4.60]
**Subtotal (95% CI)**			**609**			**555**	**100.0%**	**0.83 [0.00, 1.65]**
Heterogeneity: Tau² = 0.00; Chi² = 5.72, df = 6 (P = 0.46); I² = 0%								
Test for overall effect: Z = 1.97 (P = 0.05)								

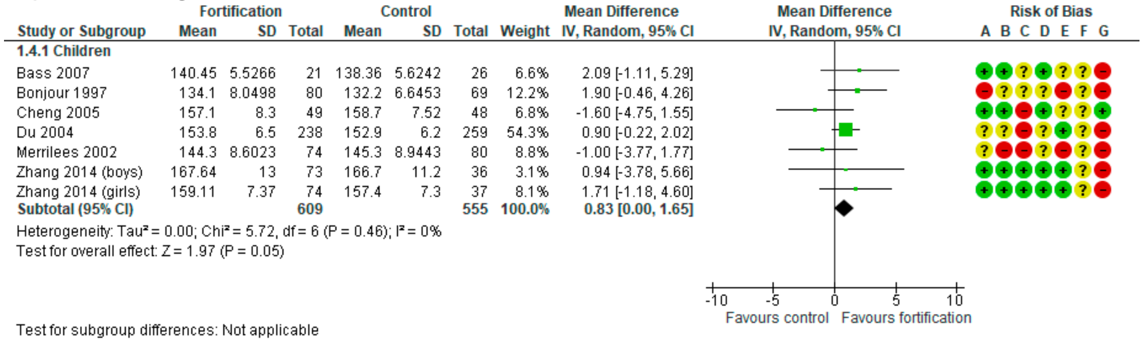

Test for subgroup differences: Not applicable

Figure S4: Parathyroid hormone (pmol/L)

	Fortification			Control				Mean Difference
**Study or Subgroup**	**Mean**	**SD**	**Total**	**Mean**	**SD**	**Total**	**Weight**	**IV, Random, 95% CI**
**1.7.1 Children**								
Du 2004 (1)	6.68	3.03	238	8.19	6.3	259	100.0%	-1.51 [-2.37, -0.65]
**Subtotal (95% CI)**			**238**			**259**	**100.0%**	**-1.51 [-2.37, -0.65]**
Heterogeneity: Not applicable								
Test for overall effect: Z = 3.45 (P = 0.0006)								
**1.7.2 Adults**								
Ferrar 2011	3.4455	1.2164	56	3.3436	1.614	20	100.0%	0.10 [-0.67, 0.88]
**Subtotal (95% CI)**			**56**			**20**	**100.0%**	**0.10 [-0.67, 0.88]**
Heterogeneity: Not applicable								
Test for overall effect: Z = 0.26 (P = 0.80)								
**1.7.3 Postmenopausal Women**								
Green 2002	3.12	1.18	38	3.4	1.28	38	100.0%	-0.28 [-0.83, 0.27]
**Subtotal (95% CI)**			**38**			**38**	**100.0%**	**-0.28 [-0.83, 0.27]**
Heterogeneity: Not applicable								
Test for overall effect: Z = 0.99 (P = 0.32)								

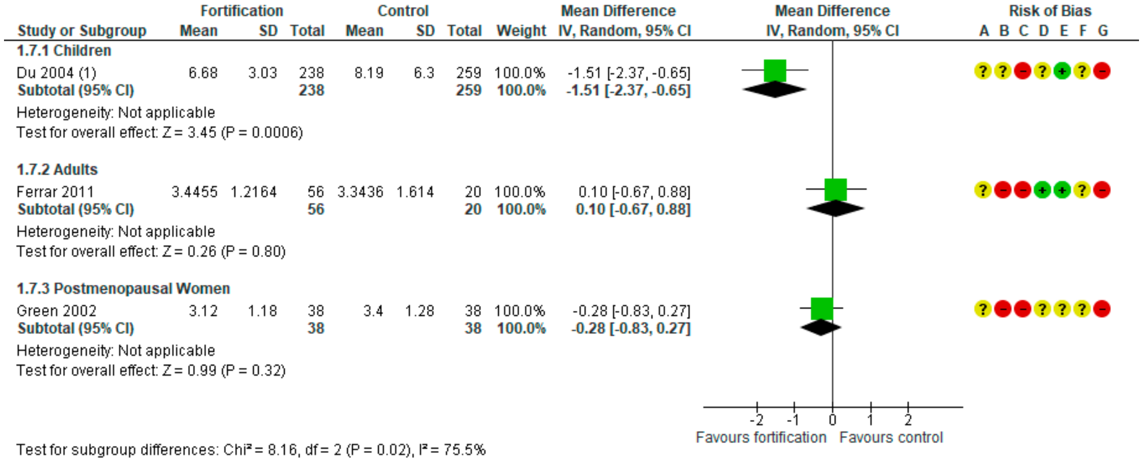

Test for subgroup differences: Chi² = 8.16, df = 2 (P = 0.02), I² = 75.5%

Figure S5: 25-hydroxy cholecalciferol (25 (OH) D3) (ng/mL)

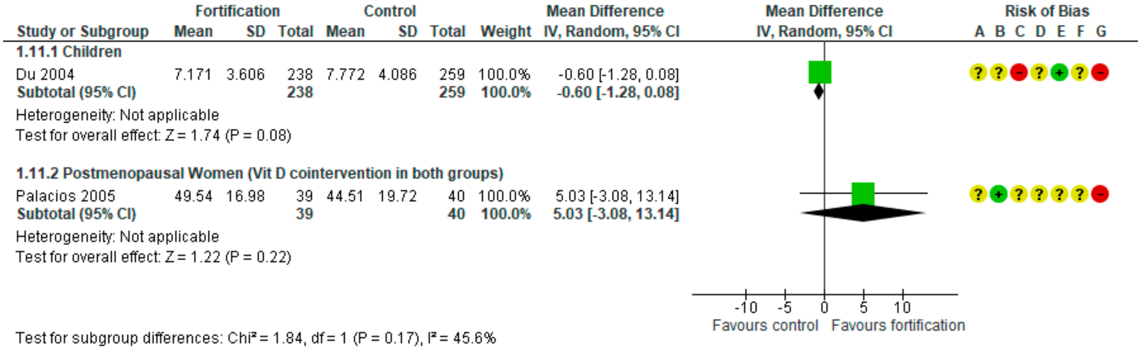

Figure S6: Serum Calcium (mg/dL)

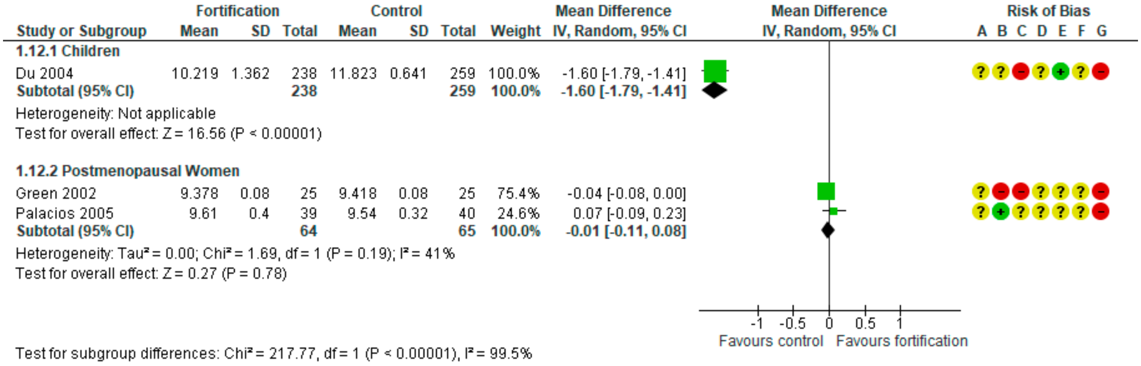

Figure S7: Urine Calcium / creatinine ratio

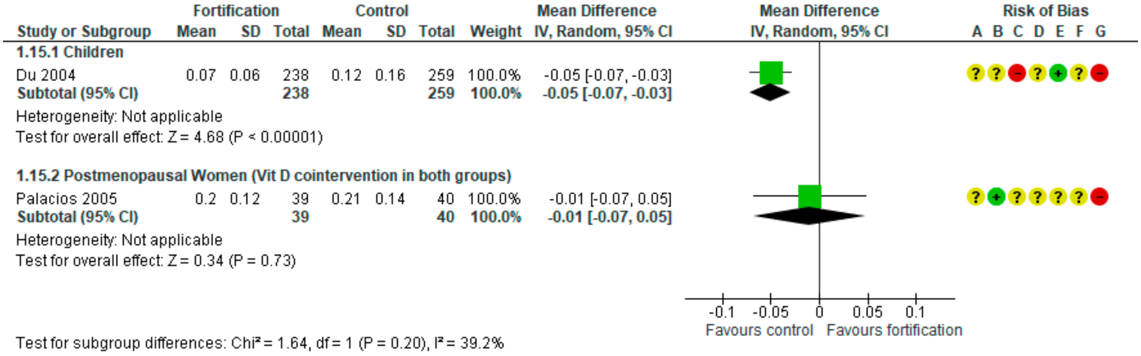

Figure S8: BMD Lumbar spine (g/cm²)

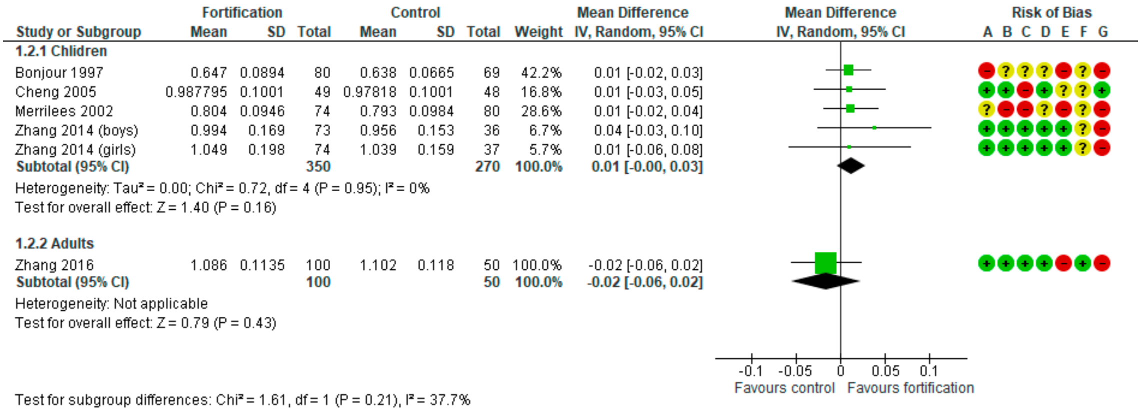

Figure S9: BMD Lumbar spine (g/cm²)

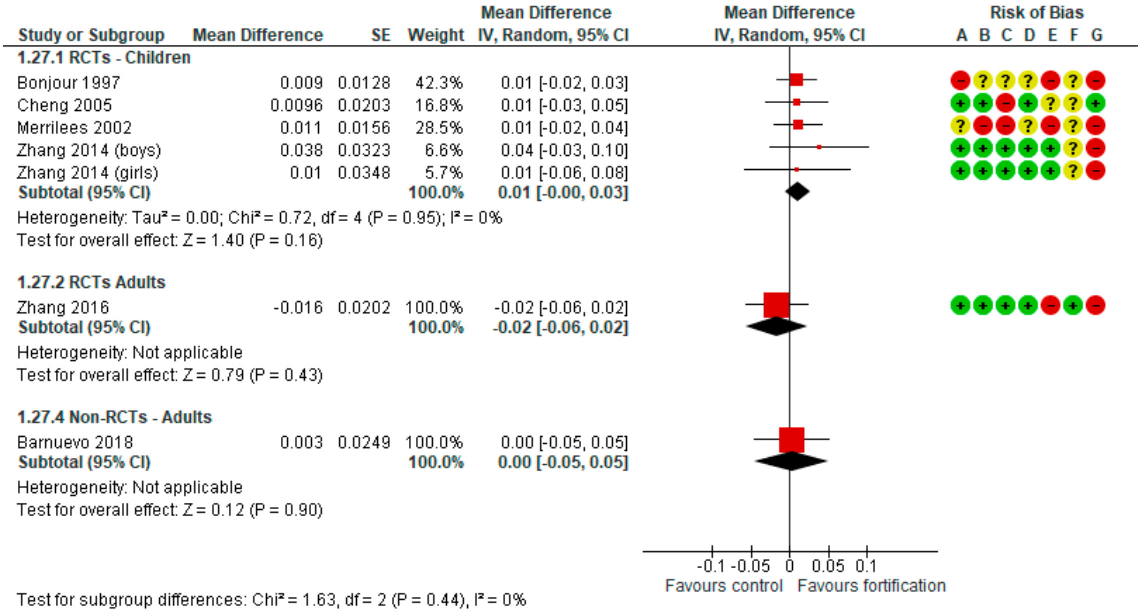

Figure S10: BMD hip (g/cm²)

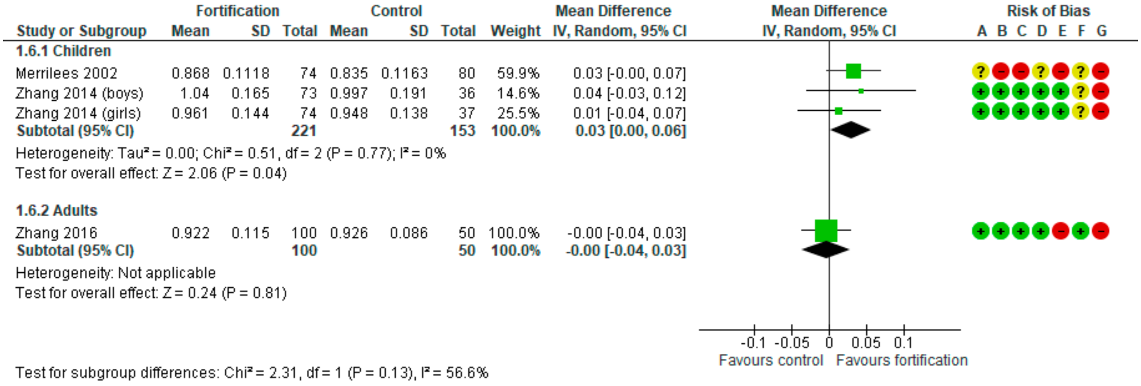

Figure S11: BMD Hip (g/cm²)

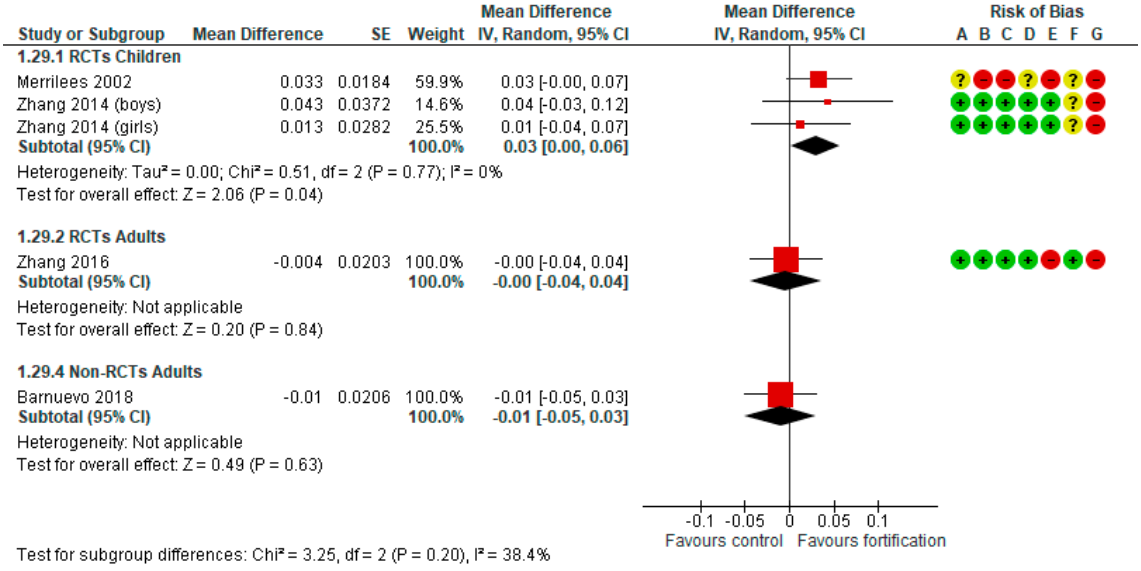

Figure S12: BMD total body (g/cm²)

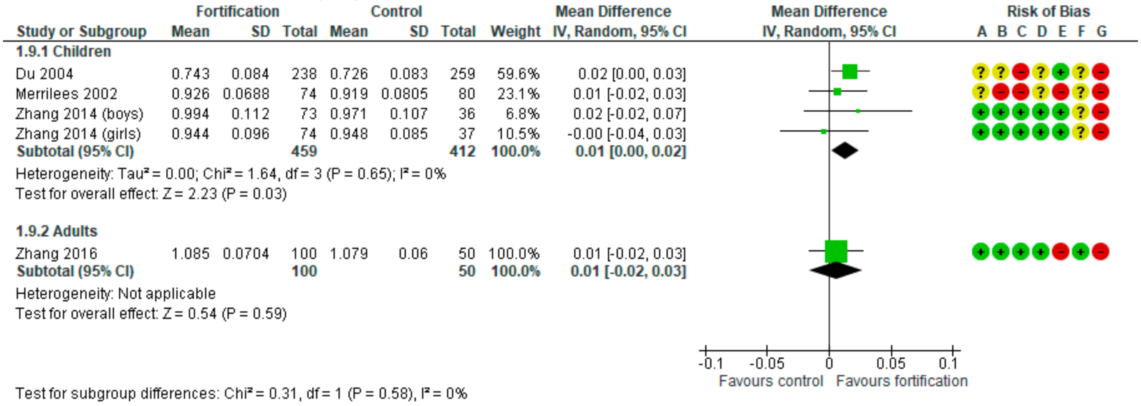

Figure S13: BMC total body (g)

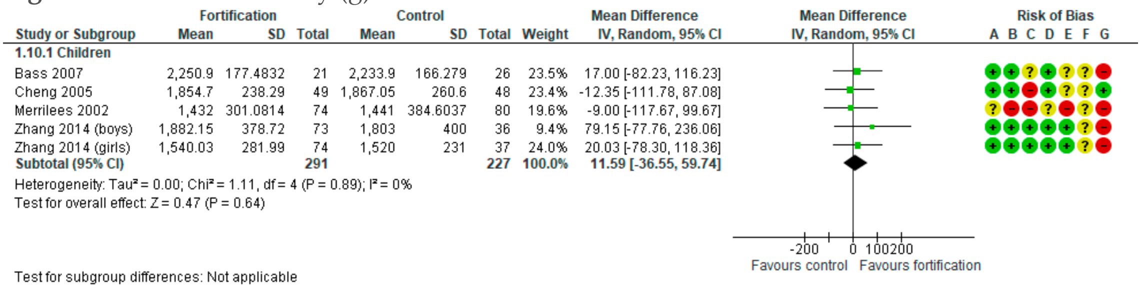

Figure S14: BMC lumbar spine (g)

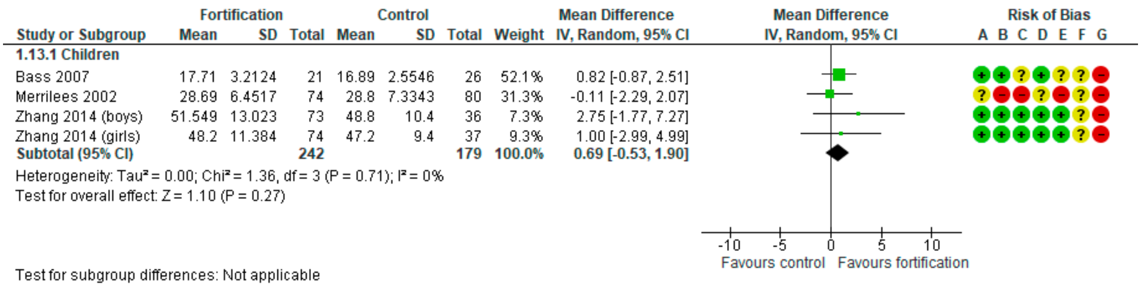

Figure S15: BMD Throcanteric region (g/cm²)

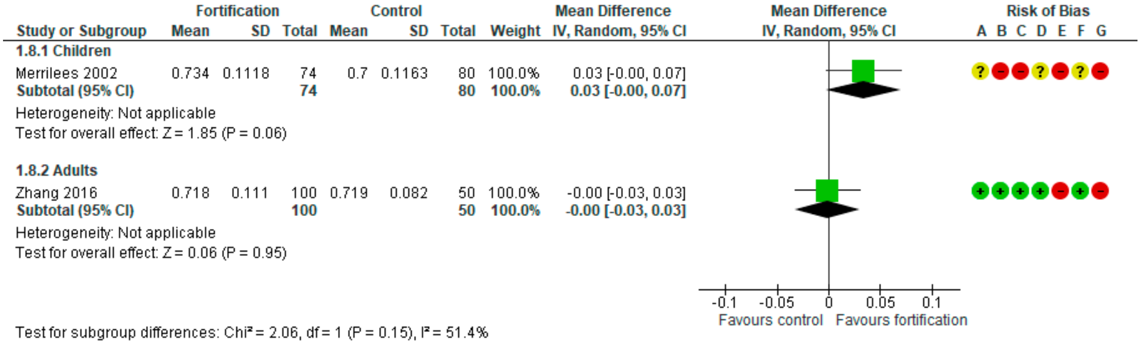

Figure S16: BMD Trochanteric region (g/cm²)

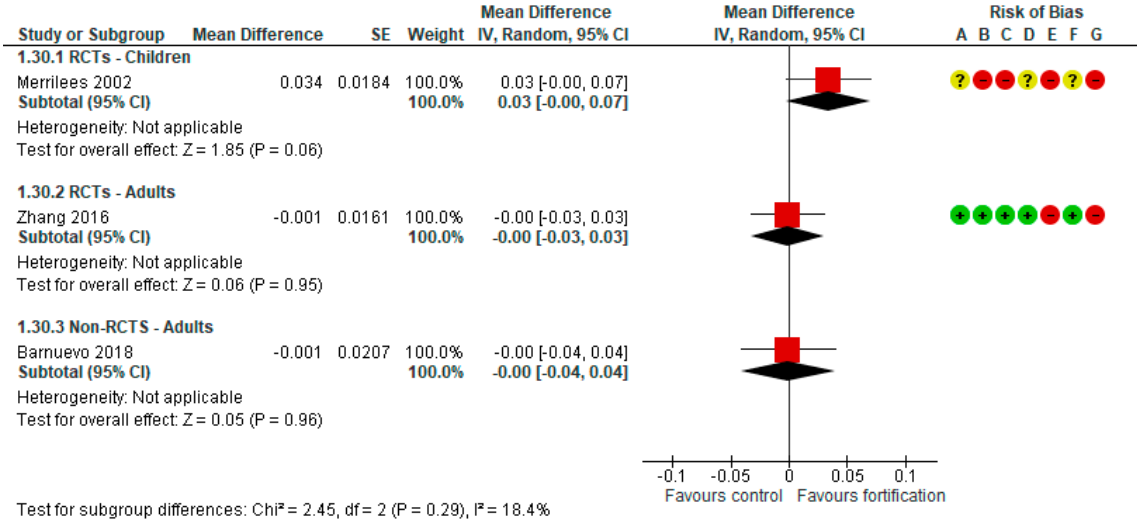

Figure S17: Calcium Intake (mg/day)

Study or Subgroup	Fortification Mean	Fortification SD	Fortification Total	Control Mean	Control SD	Control Total	Weight	Mean Difference IV, Random, 95% CI
**1.1.1 Children**								
Bass 2007	1,330	412.4318	21	934	285.5451	26	14.3%	396.00 [188.24, 603.76]
Du 2004	649.2	173.4	238	457.5	197.3	259	30.1%	191.70 [159.10, 224.30]
Zhang 2014 (boys)	1,066	208	73	729	135	36	27.8%	337.00 [272.03, 401.97]
Zhang 2014 (girls)	1,008	222	74	655	126	37	27.8%	353.00 [288.14, 417.86]
**Subtotal (95% CI)**			**406**			**358**	**100.0%**	**306.17 [198.97, 413.38]**
Heterogeneity: Tau² = 9665.77; Chi² = 30.65, df = 3 (P < 0.00001); I² = 90%								
Test for overall effect: Z = 5.60 (P < 0.00001)								
**1.1.2 Adults**								
Ferrar 2011	1,191.60714	348.42	56	873.5	442.3	20	27.5%	318.11 [103.86, 532.36]
Kukuljan 2009	1,590	301.32	45	915	286.09	44	34.4%	675.00 [552.95, 797.05]
Zhang 2016	1,167	221.284702	100	769	131	50	38.1%	398.00 [341.44, 454.56]
**Subtotal (95% CI)**			**201**			**114**	**100.0%**	**471.47 [266.51, 676.42]**
Heterogeneity: Tau² = 27870.87; Chi² = 17.60, df = 2 (P = 0.0002); I² = 89%								
Test for overall effect: Z = 4.51 (P < 0.00001)								
**1.1.3 Postmenopausal Women**								
Green 2002	2,020	80	25	810	90	25	100.0%	1210.00 [1162.80, 1257.20]
**Subtotal (95% CI)**			**25**			**25**	**100.0%**	**1210.00 [1162.80, 1257.20]**
Heterogeneity: Not applicable								
Test for overall effect: Z = 50.24 (P < 0.00001)								

Test for subgroup differences: Chi² = 259.37, df = 2 (P < 0.00001), I² = 99.2%

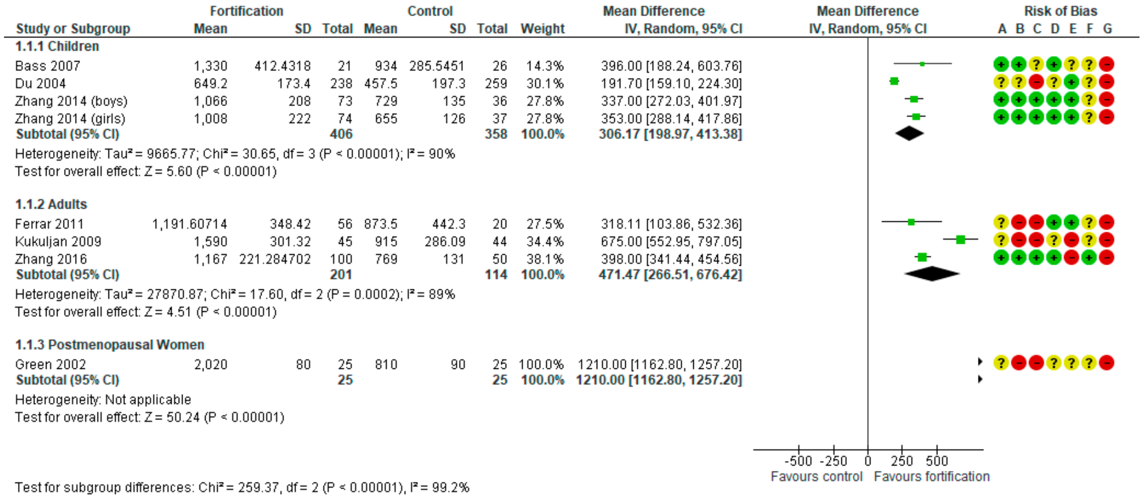

Figure S18: BMD Femoral neck (g/cm²)

Study or Subgroup	Fortification Mean	Fortification SD	Fortification Total	Control Mean	Control SD	Control Total	Weight	Mean Difference IV, Random, 95% CI
**1.5.1 Children**								
Bonjour 1997	0.656	0.0984	80	0.635	0.0831	69	39.4%	0.02 [-0.01, 0.05]
Cheng 2005	0.935	0.101	49	0.909	0.101	48	20.7%	0.03 [-0.01, 0.07]
Merrilees 2002	0.877	0.129	74	0.847	0.1163	80	22.1%	0.03 [-0.01, 0.07]
Zhang 2014 (boys)	1.009	0.166	73	0.966	0.19	36	6.3%	0.04 [-0.03, 0.12]
Zhang 2014 (girls)	0.943	0.151	74	0.933	0.129	37	11.5%	0.01 [-0.04, 0.06]
**Subtotal (95% CI)**			**350**			**270**	**100.0%**	**0.02 [0.01, 0.04]**
Heterogeneity: Tau² = 0.00; Chi² = 0.66, df = 4 (P = 0.96); I² = 0%								
Test for overall effect: Z = 2.59 (P = 0.010)								
**1.5.2 Adults**								
Zhang 2016	0.8945	0.116	100	0.901	0.081	50	100.0%	-0.01 [-0.04, 0.03]
**Subtotal (95% CI)**			**100**			**50**	**100.0%**	**-0.01 [-0.04, 0.03]**
Heterogeneity: Not applicable								
Test for overall effect: Z = 0.40 (P = 0.69)								

Test for subgroup differences: Chi² = 2.66, df = 1 (P = 0.10), I² = 62.4%

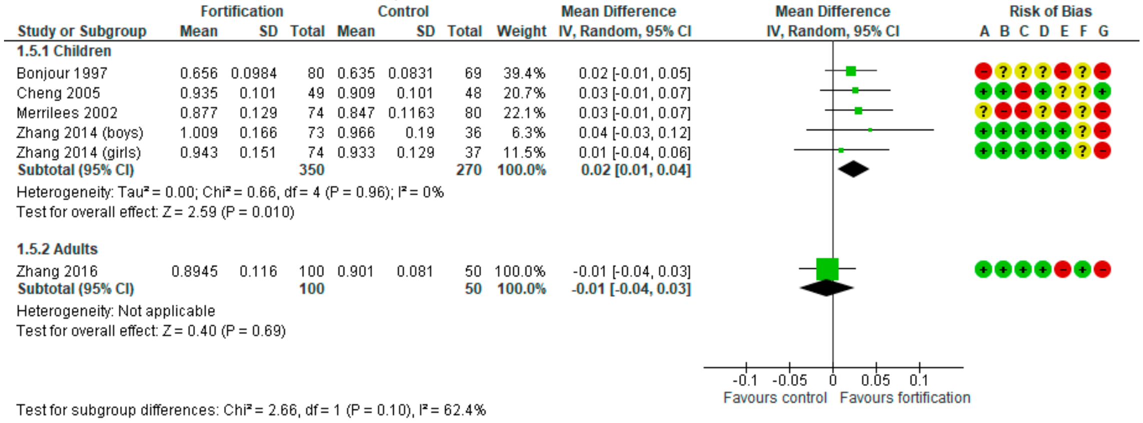

Figure S19: Body Mass Index (kg/m2)

Study or Subgroup	Fortification Mean	Fortification SD	Fortification Total	Control Mean	Control SD	Control Total	Weight	Mean Difference IV, Random, 95% CI
**1.14.1 Children**								
Bonjour 1997	16.8	1.7889	80	17.4	2.492	69	48.2%	-0.60 [-1.31, 0.11]
Du 2004	19.1	3.2	238	18.5	3	259	51.8%	0.60 [0.05, 1.15]
**Subtotal (95% CI)**			**318**			**328**	**100.0%**	**0.02 [-1.15, 1.20]**
Heterogeneity: Tau² = 0.62; Chi² = 6.93, df = 1 (P = 0.008); I² = 86%								
Test for overall effect: Z = 0.04 (P = 0.97)								

Test for subgroup differences: Not applicable

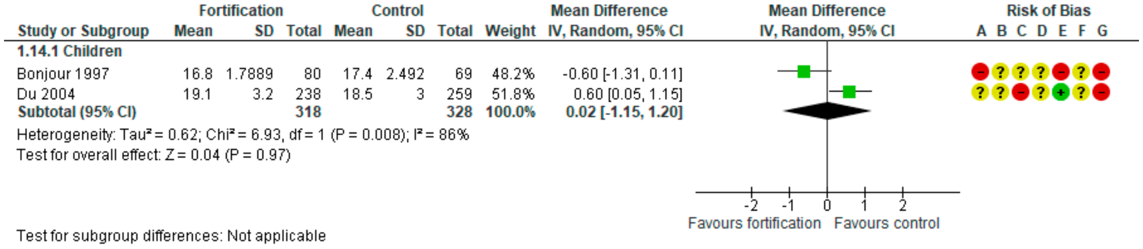

Figure S20: BMC Hip (g)

Study or Subgroup	Fortification Mean	Fortification SD	Fortification Total	Control Mean	Control SD	Control Total	Weight	Mean Difference IV, Random, 95% CI
**1.16.1 Children**								
Merrilees 2002	20.69	4.2151	74	20.41	5.0982	80	51.8%	0.28 [-1.19, 1.75]
Zhang 2014 (boys)	32.02	7.22	73	30.8	8	36	11.7%	1.22 [-1.87, 4.31]
Zhang 2014 (girls)	25.457	5.071	74	24.7	4.1	37	36.5%	0.76 [-1.00, 2.51]
**Subtotal (95% CI)**			**221**			**153**	**100.0%**	**0.56 [-0.50, 1.62]**

Heterogeneity: Tau² = 0.00; Chi² = 0.36, df = 2 (P = 0.83); I^2 = 0%
Test for overall effect: Z = 1.04 (P = 0.30)

Test for subgroup differences: Not applicable

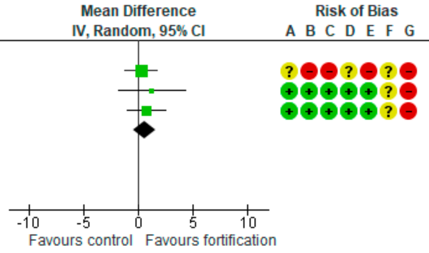

Figure S21: BMC Femoral Neck (g)

Study or Subgroup	Fortification Mean	Fortification SD	Fortification Total	Control Mean	Control SD	Control Total	Weight	Mean Difference IV, Random, 95% CI
**1.17.1 Children**								
Merrilees 2002	3.49	0.6882	74	3.4	0.7155	80	50.3%	0.09 [-0.13, 0.31]
Zhang 2014 (boys)	4.725	1.02	73	4.66	1.2	36	11.9%	0.06 [-0.39, 0.52]
Zhang 2014 (girls)	3.99	0.78	74	3.82	0.57	37	37.9%	0.17 [-0.09, 0.43]
**Subtotal (95% CI)**			**221**			**153**	**100.0%**	**0.12 [-0.04, 0.27]**

Heterogeneity: Tau² = 0.00; Chi² = 0.27, df = 2 (P = 0.87); I^2 = 0%
Test for overall effect: Z = 1.46 (P = 0.14)

Test for subgroup differences: Not applicable

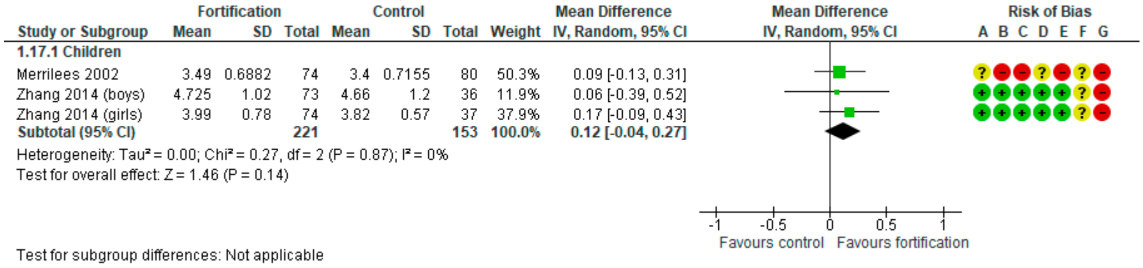

Figure S22: BMC Femoral Shaft (g)

Study or Subgroup	Fortification Mean	Fortification SD	Fortification Total	Control Mean	Control SD	Control Total	Weight	Mean Difference IV, Random, 95% CI
**1.21.1 Children**								
Zhang 2014 (boys)	16.556	3.439	73	15.9	3.7	36	31.8%	0.66 [-0.79, 2.10]
Zhang 2014 (girls)	14.22	2.787	74	13.99	2.34	37	68.2%	0.23 [-0.76, 1.22]
**Subtotal (95% CI)**			**147**			**73**	**100.0%**	**0.37 [-0.45, 1.18]**

Heterogeneity: Tau² = 0.00; Chi² = 0.23, df = 1 (P = 0.63); I^2 = 0%
Test for overall effect: Z = 0.88 (P = 0.38)

Test for subgroup differences: Not applicable

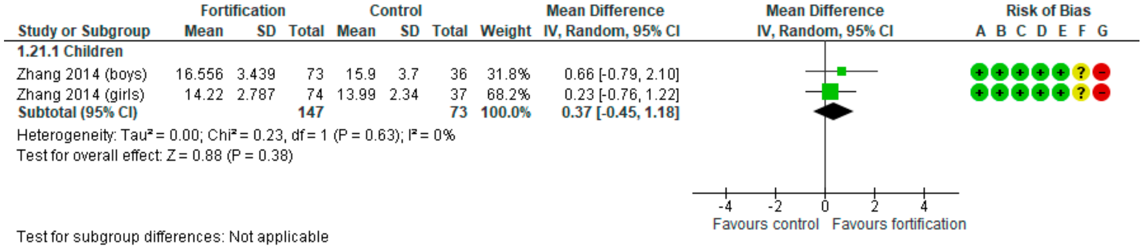

Figure S23: BMD Femoral Shaft (g/cm²)

Study or Subgroup	Fortification Mean	Fortification SD	Fortification Total	Control Mean	Control SD	Control Total	Weight	Mean Difference IV, Random, 95% CI
Zhang 2014 (boys)	1.17	0.197	73	1.119	0.227	36	38.1%	0.05 [-0.04, 0.14]
Zhang 2014 (girls)	1.105	0.187	74	1.084	0.165	37	61.9%	0.02 [-0.05, 0.09]
**Total (95% CI)**			**147**			**73**	**100.0%**	**0.03 [-0.02, 0.09]**

Heterogeneity: Tau² = 0.00; Chi² = 0.28, df = 1 (P = 0.59); I^2 = 0%
Test for overall effect: Z = 1.19 (P = 0.24)

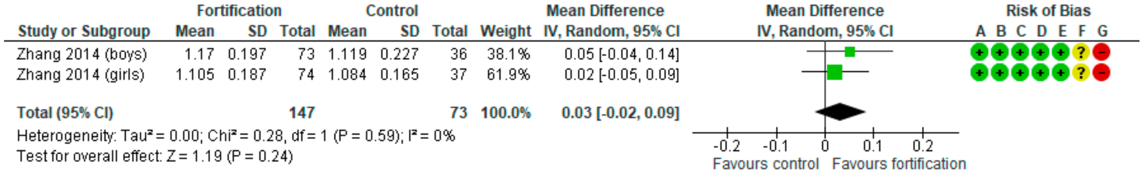

Figure S24: Parathyroid hormone by calcium fortification level (pmol/L)

Study or Subgroup	Fortification Mean	Fortification SD	Fortification Total	Control Mean	Control SD	Control Total	Weight	Mean Difference IV, Random, 95% CI
**1.43.1 Low fortification 244 mg/day**								
Ferrar 2011	31.32	11.38	19	31.53	15.22	20	100.0%	-0.21 [-8.62, 8.20]
**Subtotal (95% CI)**			**19**			**20**	**100.0%**	**-0.21 [-8.62, 8.20]**
Heterogeneity: Not applicable								
Test for overall effect: Z = 0.05 (P = 0.96)								
**1.43.2 Medium fortification 459 mg/day**								
Ferrar 2011	35.06	13.63	17	31.53	15.22	20	100.0%	3.53 [-5.77, 12.83]
**Subtotal (95% CI)**			**17**			**20**	**100.0%**	**3.53 [-5.77, 12.83]**
Heterogeneity: Not applicable								
Test for overall effect: Z = 0.74 (P = 0.46)								
**1.43.3 High fortification 676 mg/day**								
Ferrar 2011	31.42	9.66	20	31.53	15.22	20	100.0%	-0.11 [-8.01, 7.79]
**Subtotal (95% CI)**			**20**			**20**	**100.0%**	**-0.11 [-8.01, 7.79]**
Heterogeneity: Not applicable								
Test for overall effect: Z = 0.03 (P = 0.98)								

Test for subgroup differences: Chi² = 0.44, df = 2 (P = 0.80), I² = 0%

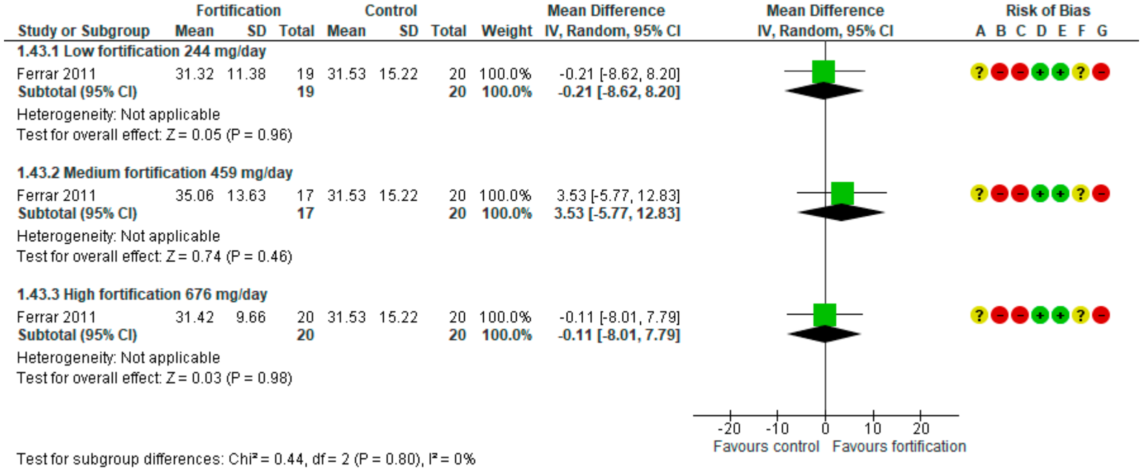

Figure S25: Serum 1,25-dihydroxycholecalciferol by calcium fortification level (nmol/l)

Study or Subgroup	Fortification Mean	Fortification SD	Fortification Total	Control Mean	Control SD	Control Total	Weight	Mean Difference IV, Random, 95% CI
**1.44.1 Low fortification 244 mg/day**								
Ferrar 2011	150.74	65.05	19	150.96	54.55	20	100.0%	-0.22 [-38.00, 37.56]
**Subtotal (95% CI)**			**19**			**20**	**100.0%**	**-0.22 [-38.00, 37.56]**
Heterogeneity: Not applicable								
Test for overall effect: Z = 0.01 (P = 0.99)								
**1.44.2 Medium fortification 459 mg/day**								
Ferrar 2011	140.21	71.4	17	150.96	54.55	20	100.0%	-10.75 [-52.27, 30.77]
**Subtotal (95% CI)**			**17**			**20**	**100.0%**	**-10.75 [-52.27, 30.77]**
Heterogeneity: Not applicable								
Test for overall effect: Z = 0.51 (P = 0.61)								
**1.44.3 High fortification 676 mg/day**								
Ferrar 2011	150.47	38.96	20	150.96	54.55	20	100.0%	-0.49 [-29.87, 28.89]
**Subtotal (95% CI)**			**20**			**20**	**100.0%**	**-0.49 [-29.87, 28.89]**
Heterogeneity: Not applicable								
Test for overall effect: Z = 0.03 (P = 0.97)								

Test for subgroup differences: Chi² = 0.18, df = 2 (P = 0.91), I² = 0%

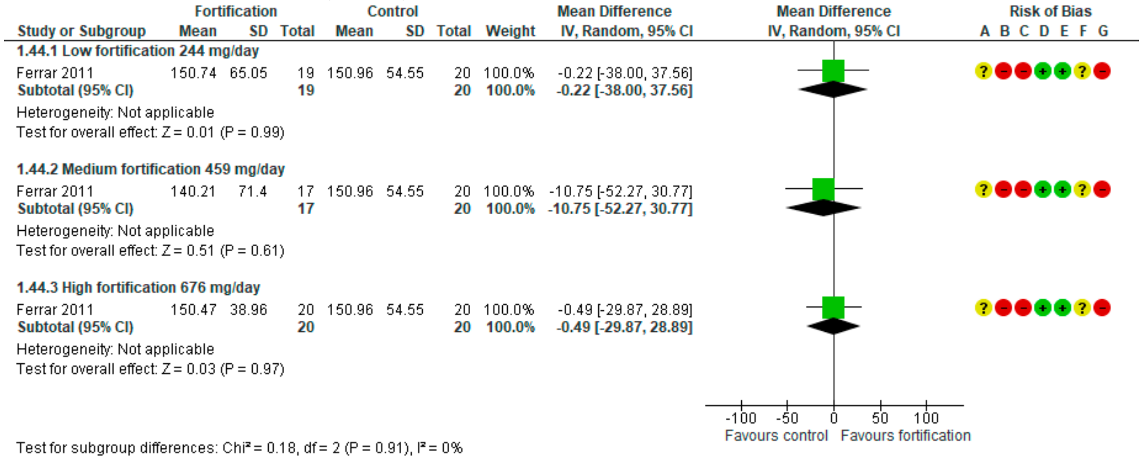

Figure S26: BMD Lumbar spine by calcium fortification level (g/cm²)

Study or Subgroup	Fortification Mean	Fortification SD	Fortification Total	Control Mean	Control SD	Control Total	Weight	Mean Difference IV, Random, 95% CI
**1.45.1 Medium fortification 600 mg/day**								
Zhang 2014 (boys)	1.006	0.185	38	0.956	0.153	36	20.7%	0.05 [-0.03, 0.13]
Zhang 2014 (girls)	1.044	0.206	36	1.039	0.159	37	17.3%	0.01 [-0.08, 0.09]
Zhang 2016	1.087	0.102	50	1.102	0.118	50	61.9%	-0.02 [-0.06, 0.03]
**Subtotal (95% CI)**			**124**			**123**	**100.0%**	**0.00 [-0.03, 0.04]**
Heterogeneity: Tau² = 0.00; Chi² = 2.08, df = 2 (P = 0.35); I² = 4%								
Test for overall effect: Z = 0.11 (P = 0.92)								
**1.45.2 High fortification 900 mg/day**								
Zhang 2014 (boys)	0.981	0.152	35	0.956	0.153	36	24.9%	0.03 [-0.05, 0.10]
Zhang 2014 (girls)	1.055	0.192	38	1.039	0.159	37	19.8%	0.02 [-0.06, 0.10]
Zhang 2016	1.086	0.125	50	1.102	0.118	50	55.3%	-0.02 [-0.06, 0.03]
**Subtotal (95% CI)**			**123**			**123**	**100.0%**	**0.00 [-0.03, 0.04]**
Heterogeneity: Tau² = 0.00; Chi² = 1.06, df = 2 (P = 0.59); I² = 0%								
Test for overall effect: Z = 0.03 (P = 0.98)								

Test for subgroup differences: Chi² = 0.00, df = 1 (P = 0.96), I² = 0%

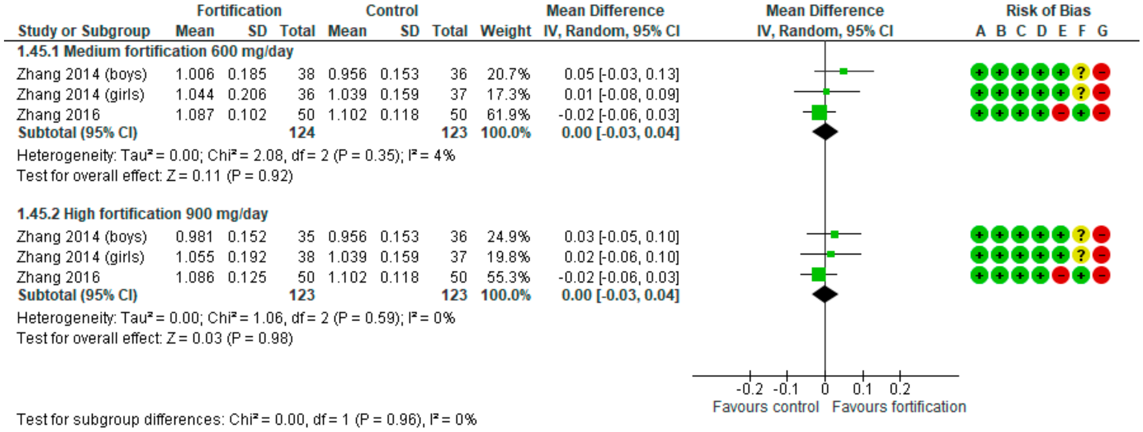

Figure S27: Weight by calcium fortification level (Kg)

Study or Subgroup	Fortification Mean	SD	Total	Control Mean	SD	Total	Weight	Mean Difference IV, Random, 95% CI
**1.46.1 Medium fortification 600 mg/day**								
Zhang 2014 (boys)	59.1	18.5	38	53.2	16.9	36	9.9%	5.90 [-2.17, 13.97]
Zhang 2014 (girls)	49.2	9.6	36	48.1	9.9	37	32.3%	1.10 [-3.37, 5.57]
Zhang 2016	50.9	9.6	50	50.9	7.3	50	57.8%	0.00 [-3.34, 3.34]
**Subtotal (95% CI)**			**124**			**123**	**100.0%**	**0.94 [-1.60, 3.48]**
Heterogeneity: Tau² = 0.00; Chi² = 1.76, df = 2 (P = 0.41); I² = 0%								
Test for overall effect: Z = 0.73 (P = 0.47)								
**1.46.2 High fortification 900 mg/day**								
Zhang 2014 (boys)	57.6	12.7	35	53.2	16.9	36	13.4%	4.40 [-2.54, 11.34]
Zhang 2014 (girls)	49.8	10.7	38	48.1	9.9	37	29.7%	1.70 [-2.96, 6.36]
Zhang 2016	54.6	9.7	50	50.9	7.3	50	57.0%	3.70 [0.34, 7.06]
**Subtotal (95% CI)**			**123**			**123**	**100.0%**	**3.20 [0.66, 5.74]**
Heterogeneity: Tau² = 0.00; Chi² = 0.60, df = 2 (P = 0.74); I² = 0%								
Test for overall effect: Z = 2.47 (P = 0.01)								

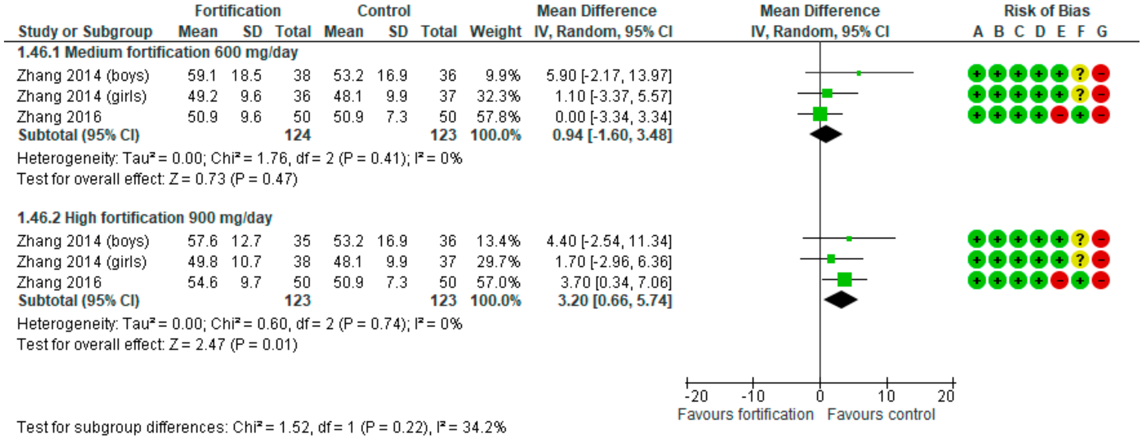

Test for subgroup differences: Chi² = 1.52, df = 1 (P = 0.22), I² = 34.2%

Figure S28: BMD Femoral neck by calcium fortification level (g/cm²)

Study or Subgroup	Fortification Mean	SD	Total	Control Mean	SD	Total	Weight	Mean Difference IV, Random, 95% CI
**1.47.1 Medium fortification 600 mg/day**								
Zhang 2014 (boys)	1.015	0.162	38	0.966	0.19	36	14.6%	0.05 [-0.03, 0.13]
Zhang 2014 (girls)	0.933	0.158	36	0.933	0.129	37	21.6%	0.00 [-0.07, 0.07]
Zhang 2016	0.902	0.113	50	0.901	0.081	50	63.8%	0.00 [-0.04, 0.04]
**Subtotal (95% CI)**			**124**			**123**	**100.0%**	**0.01 [-0.02, 0.04]**
Heterogeneity: Tau² = 0.00; Chi² = 1.18, df = 2 (P = 0.56); I² = 0%								
Test for overall effect: Z = 0.50 (P = 0.62)								
**1.47.2 High fortification 900 mg/day**								
Zhang 2014 (boys)	1.003	0.172	35	0.966	0.19	36	13.8%	0.04 [-0.05, 0.12]
Zhang 2014 (girls)	0.954	0.145	38	0.933	0.129	37	25.4%	0.02 [-0.04, 0.08]
Zhang 2016	0.887	0.12	50	0.901	0.081	50	60.8%	-0.01 [-0.05, 0.03]
**Subtotal (95% CI)**			**123**			**123**	**100.0%**	**0.00 [-0.03, 0.03]**
Heterogeneity: Tau² = 0.00; Chi² = 1.63, df = 2 (P = 0.44); I² = 0%								
Test for overall effect: Z = 0.12 (P = 0.90)								

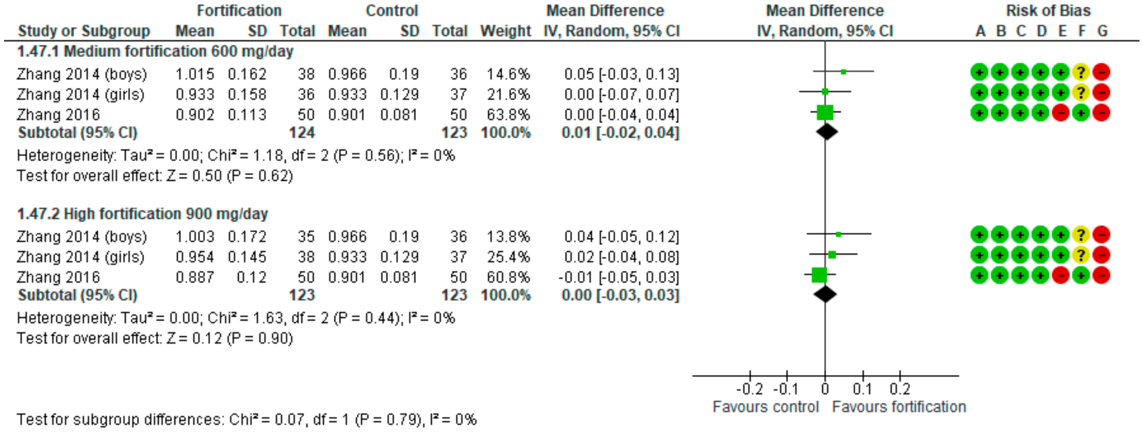

Test for subgroup differences: Chi² = 0.07, df = 1 (P = 0.79), I² = 0%

Figure S29: BMD hip by calcium fortification level (g/cm²)

Study or Subgroup	Fortification Mean	SD	Total	Control Mean	SD	Total	Weight	Mean Difference IV, Random, 95% CI
**1.48.1 Medium fortification 600 mg/day**								
Zhang 2014 (boys)	1.055	0.163	38	0.997	0.191	36	13.5%	0.06 [-0.02, 0.14]
Zhang 2014 (girls)	0.954	0.153	36	0.948	0.138	37	19.9%	0.01 [-0.06, 0.07]
Zhang 2016	0.931	0.1	50	0.926	0.086	50	66.6%	0.01 [-0.03, 0.04]
**Subtotal (95% CI)**			**124**			**123**	**100.0%**	**0.01 [-0.02, 0.04]**
Heterogeneity: Tau² = 0.00; Chi² = 1.41, df = 2 (P = 0.49); I² = 0%								
Test for overall effect: Z = 0.81 (P = 0.42)								
**1.48.2 High fortification 900 mg/day**								
Zhang 2014 (boys)	1.024	0.168	35	0.997	0.191	36	15.1%	0.03 [-0.06, 0.11]
Zhang 2014 (girls)	0.967	0.138	38	0.948	0.138	37	27.1%	0.02 [-0.04, 0.08]
Zhang 2016	0.913	0.128	50	0.926	0.086	50	57.8%	-0.01 [-0.06, 0.03]
**Subtotal (95% CI)**			**123**			**123**	**100.0%**	**0.00 [-0.03, 0.03]**
Heterogeneity: Tau² = 0.00; Chi² = 1.10, df = 2 (P = 0.58); I² = 0%								
Test for overall effect: Z = 0.10 (P = 0.92)								

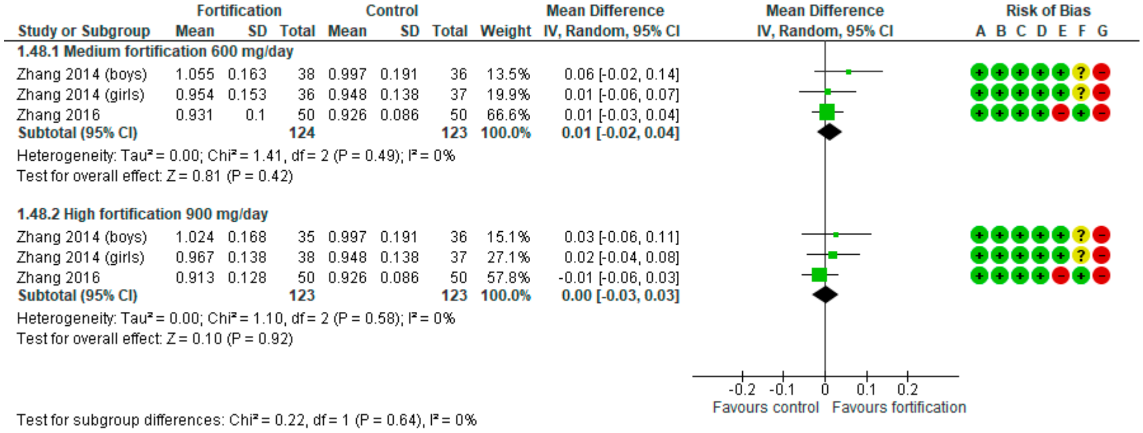

Test for subgroup differences: Chi² = 0.22, df = 1 (P = 0.64), I² = 0%

Figure S30: BMD total body by calcium fortification level (g/cm²)

Study or Subgroup	Fortification Mean	Fortification SD	Fortification Total	Control Mean	Control SD	Control Total	Weight	Mean Difference IV, Random, 95% CI
**1.49.1 Medium fortification 600 mg/day**								
Zhang 2014 (boys)	1.001	0.125	38	0.971	0.107	36	13.1%	0.03 [-0.02, 0.08]
Zhang 2014 (girls)	0.942	0.1	36	0.948	0.085	37	20.3%	-0.01 [-0.05, 0.04]
Zhang 2016	1.087	0.06	50	1.079	0.06	50	66.6%	0.01 [-0.02, 0.03]
**Subtotal (95% CI)**			**124**			**123**	**100.0%**	**0.01 [-0.01, 0.03]**
Heterogeneity: Tau² = 0.00; Chi² = 1.08, df = 2 (P = 0.58); I² = 0%								
Test for overall effect: Z = 0.82 (P = 0.41)								
**1.49.2 High fortification 900 mg/day**								
Zhang 2014 (boys)	0.987	0.097	35	0.971	0.107	36	18.8%	0.02 [-0.03, 0.06]
Zhang 2014 (girls)	0.947	0.093	38	0.948	0.085	37	26.1%	-0.00 [-0.04, 0.04]
Zhang 2016	1.083	0.08	50	1.079	0.06	50	55.1%	0.00 [-0.02, 0.03]
**Subtotal (95% CI)**			**123**			**123**	**100.0%**	**0.00 [-0.02, 0.03]**
Heterogeneity: Tau² = 0.00; Chi² = 0.30, df = 2 (P = 0.86); I² = 0%								
Test for overall effect: Z = 0.47 (P = 0.64)								

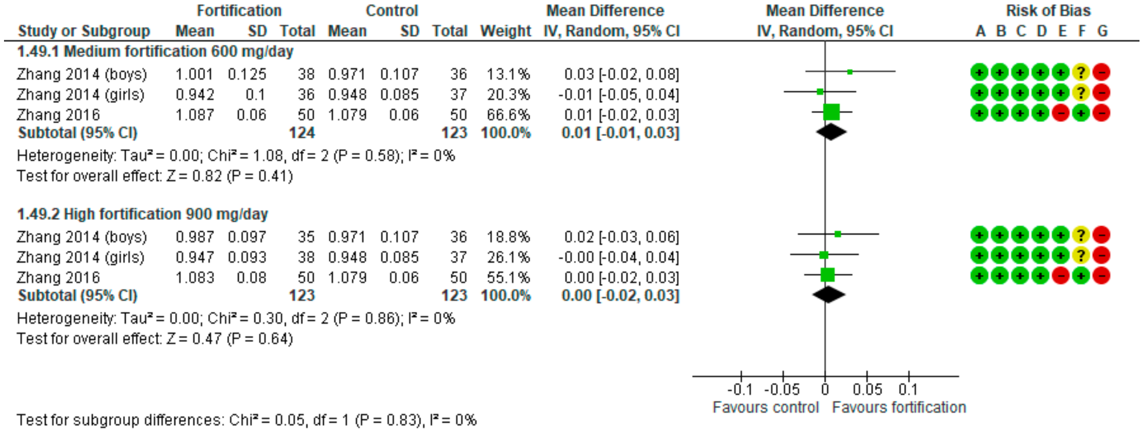

Test for subgroup differences: Chi² = 0.05, df = 1 (P = 0.83), I² = 0%
